# Real-Time Monitoring and Step-by-Step Guidance for Transcatheter Tricuspid Annuloplasty Using Transesophageal Echocardiography

**DOI:** 10.3390/jcdd9120415

**Published:** 2022-11-25

**Authors:** Yu Liu, Wei Li, Daxin Zhou, Xiaochun Zhang, Dehong Kong, Zhenyi Ge, Haiyan Chen, Xianhong Shu, Cuizhen Pan, Junbo Ge

**Affiliations:** 1 Shanghai Institute of Medical Imaging, Shanghai 200032, China; 2 Department of Echocardiography, Zhongshan Hospital, Fudan University, Shanghai 200032, China; 3 Shanghai Institute of Cardiovascular Diseases, Shanghai 200032, China; 4 National Clinical Research Center for Interventional Therapy, Shanghai 200032, China; 5 Department of Cardiology, Zhongshan Hospital, Fudan University, Shanghai 200032, China

**Keywords:** tricuspid regurgitation, tricuspid annuloplasty, transesophageal echocardiography, tricuspid valve intervention

## Abstract

Transcatheter tricuspid valve intervention (TTVI) is a novel alternative to functional tricuspid regurgitation (FTR) for patients with prohibitive surgical risk. Devices have been designed according to different pathophysiological mechanisms of FTR, including ones to achieve an edge-to-edge repair and others aiming at direct annuloplasty. Recently, a transcatheter tricuspid valve repair system mimicking a surgical Kay procedure (K-Clip™ system, Huihe Medical Technology, Shanghai, China) completed its salvage-use trial. The system, which clips the posterior annulus to achieve bicuspidization of the TV, demonstrated acceptable procedural safety and efficacy. Each TTVI system has distinct characteristics for echocardiographic imaging and special consideration for intraoperative guidance. This review focuses on elaborating how two-dimensional and three-dimensional transesophageal echocardiography (TEE) are used in clinical practice to guide K-Clip™ implantation in comparison to other direct annular reduction devices. A limited number of TEE work planes are proposed for the procedure with the aim to provide a steeper learning curve for the echocardiographer and interventionalist while simplifying the implantation steps.

## 1. Introduction

Significant—moderate or severe—tricuspid regurgitation (TR) is independently associated with an adverse prognosis across different patient populations and comorbidities [1]. Current guidelines recommend surgical correction of severe TR in symptomatic patients with right ventricular (RV) dilatation and preserved RV function [2,3]. However, isolated tricuspid valve surgery is rarely performed based on its reported high mortality rates [4,5].

Transcatheter tricuspid valve intervention (TTVI) is a novel alternative for patients with prohibitive surgical risk [6]. Three TTVI devices targeting different pathophysiological mechanisms of functional TR (FTR) are available [7,8,9]. TriClip and PASCAL systems are designed to achieve a percutaneous edge-to-edge repair of the tricuspid valve (TV) and restore leaflet coaptation. The Cardioband system reduces the tricuspid annular dimension from the anteroseptal through the posteroseptal commissure and thus mimics surgical annuloplasty. As tricuspid annular dilatation mainly occurs posteriorly along the free wall aspect [10,11], devices that reduce the posterior annular dimension alone may also provide effective annular reduction [12].

**Figure 1:**
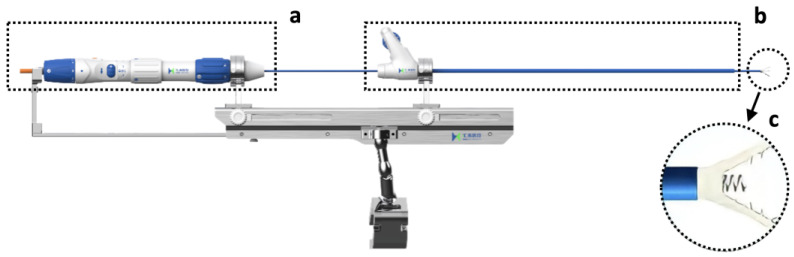
The K-Clip^TM^ system. (**a**) The delivery module. (**b**) The guide sheath and its corresponding control module. (**c**) The clamp arms and the tapping screw in between.

A new TTVI system was designed to replicate a surgical Kay procedure and achieve posterior annular reduction and biscuspidization of the TV (Figure 1, K-Clip™ system, Huihe Medical Technology, Shanghai, China). The system started its first-in-human (FIM) study (Figure S1) [13], completed enrollment (19 patients received device implantation, Tables S1 and S2), and demonstrated acceptable procedural safety and efficacy (Figures S2 and S3, Table S3).

Transesophageal echocardiography (TEE) plays an important role in the preoperative screening and planning, intraoperative guidance, and immediate postoperative evaluation of TTVI. Assessment and grading of TR before and after a transcatheter intervention have established recommendations [14,15]. However, each TTVI system has its distinct characteristics in echocardiographic imaging and special consideration for pre-operative planning and intraoperative guidance [16].

The K-Clip™ transcatheter tricuspid valve repair system is guided mainly with echocardiography, but the intraprocedural imaging required for this procedure has yet to be described. This review explores how 2D and 3D TEE are used to guide the implantation of the K-Clip™ system and discusses the imaging requirements of this novel procedure in comparison to other TTVI annuloplasty techniques.

## 2. Transcatheter Tricuspid Annuloplasty

Annuloplasty with prosthetic rings is a fundamental step in the surgical repair of FTR [17]. Though edge-to-edge repair by a transcatheter device has been widely reported to successfully treat FTR [7,18], efforts to replicate durable surgical correction have enabled several transcatheter direct annuloplasty options: the Cardioband, TriCinch, Millepede, PASTA, Trialign, and K-Clip™. To date, only the Cardioband system has been cleared for clinical use. It should be noted that nomenclature including anteroposterior commissure and posterior annulus etc. is bound to normal tricuspid anatomy and, therefore, none of these transcatheter TV repair choices are valid for congenital diseases such as Ebstein’s anomaly and tricuspid dysplasia.

The Cardioband system consists of a polyester annuloplasty band that is implanted with anchors inserted through the septolateral tricuspid annulus (TA) from the atrial side [16]. The system is delivered through a transfemoral approach. After the anchors are placed, the size of the band can be adjusted to reduce the septolateral TA diameter under real-time TEE monitoring to obtain a satisfactory reduction of TR. The Cardioband system requires both fluoroscopy and TEE for intraprocedural guidance because a coronary wire needs to be positioned in the right coronary artery. This wire serves as a TA marker and enables the necessary assessment of the distance between the anchors and the right coronary artery.

**Figure 2:**
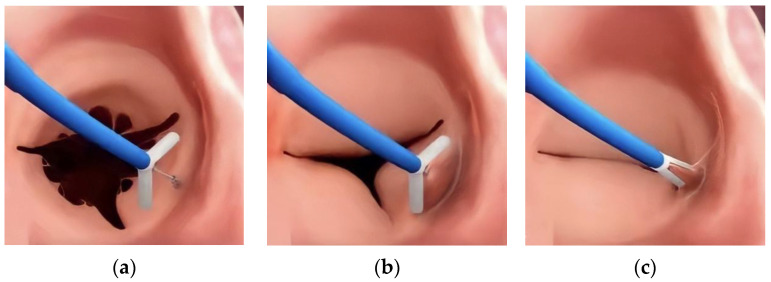
The main steps of K-Clip™ implantation. (**a**): The opening clamp and the tapping screw-shaped anchor; (**b**): the tapping screw-shaped anchor is inserted into the middle of the posterior TA and pulled back to bring the surrounding annular tissue into the clamp; (**c**): the clamp is closed to plicate the TA and achieve bicuspidization of the TV.

The Trialign and the K-Clip™ are direct annuloplasty systems that target the posterior TA and achieve TA diameter reduction through the tissue plication [19]. These systems are both delivered through a trans-jugular approach. In Trialign, the tissue plication and annular reduction are achieved through approximation of two pledgets placed at the anteroposterior and posteroseptal commissures. For the K-Clip™, a tapping screw-shaped anchor is first inserted through the middle of the posterior annulus from the atrial side and then pulled back with the surrounding annulus tissue, which is then clamped to achieve tissue plication and bicuspidization (Figure 2). For both procedures, 2D/3D TEE is needed to confirm that enough space is present on the posterior TA to either place the two pledgeted sutures or be clamped.

## 3. Echocardiographic Protocol for K-Clip™ Transcatheter Tricuspid Annuloplasty

### 3.1. Patient Selection

Patients are selected for K-Clip™ implantation based on clinical characteristics and imaging features. The clinical characteristics (Figure S1) include symptomatic severe isolated secondary TR with left ventricular ejection fraction over 40% and systolic pulmonary arterial pressure below 55 mm Hg. In addition, several imaging features of the TA and TV should be considered for the candidacy of K-Clip™ implantation.

The current K-Clip™ systems offer four choices of clip arm lengths ranging from 12 mm to 18 mm with 2 mm increments. These clip arms correspond to maximal annular reduction lengths ranging from 25 mm to 36 mm. A posterior annular dimension that falls within the range is more likely to be successfully clamped to achieve bicuspidization of the TV. A posterior TA that exceeds the longest maximal annular reduction length may not achieve meaningful TR reduction (more than one grade).

For the choice of the annular target location, the middle of the posterior annulus would be an ideal target to obtain full bicuspidization of the valve. However, when a significant coaptation gap between the anterior and posterior leaflet exists and the clip arm length can achieve sufficient plication of the posterior annulus, aiming for the anteroposterior commissure of the TA or the anterior 1/3 of the posterior annulus could achieve both annulus and coaptation gap reduction and, thus, greater reduction in tricuspid regurgitation severity. These three suggested clip implantation sites were chosen based on the analysis of the vena contracta during tricuspid regurgitation, which could be achieved either through the transgrastric projections in the short axis of TA [20] or the 3D color Doppler imaging (CDI) of the TV complex [21]. If the TR results predominantly from the coaptation gap between the anterior and posterior leaflet, the anteroposterior commissure of TA or the anterior 1/3 of the posterior annulus could be considered. Further selection between these two sites is based on whether such a target could also achieve acceptable posterior annular reduction.

The K-Clip™ system may not be a valid choice for TR that involves the septal commissures. The anteroseptal annulus is located outside of the free wall portion and thus clamping of this site may damage adjacent tissue. This target is also difficult to reach for a trans-jugular delivery system and thus is not recommended. Theoretically, the posteroseptal commissure is safe to be clamped and could be reached by the current delivery catheter, but experience with reaching this target is lacking.

### 3.2. Introducing the Delivery System (Mid-Esophageal Bicaval View)

**Figure 3:**
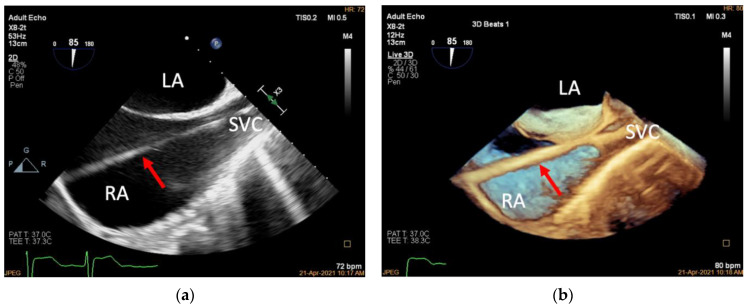
Introducing the guidewire. (**a**): Mid-esophageal bicaval view (transducer angle at 70–110°), 2D TEE guiding the Super Stiff guidewire through the superior vena cava to the inferior vena cava (red arrow); (**b**): 3D TEE of the same view. LA, left atrium; RA, right atrium; SVC, superior vena cava.

**Figure 4:**
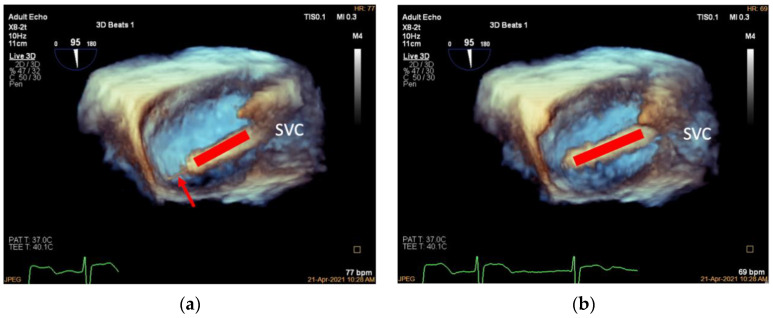
Advancing the guide sheath. (**a**): Three-dimensional mid-esophageal bicaval view, the guide sheath (red line) slowly advancing along the guidewire (red arrow) to the lower third of the right atrium at about 1 cm from the inferior vena cava; (**b**): the same view showing the guide sheath after withdrawing the Super Stiff guidewire (red line). SVC, superior vena cava.

The K-Clip™ procedure is performed in the hybrid operating room with patients under general anesthesia. Two-dimensional and 3D TEE mid-esophageal (ME) bicaval views are used to guide the Super Stiff guidewire through the superior vena cava to the inferior vena cava (Figure 3). The 18F adjustable guide sheath is advanced along the Super Stiff guidewire to the lower third of the right atrium under TEE surveillance to ensure that the end of the guide sheath is located approximately 1 cm from the inferior vena cava orifice in the right atrium (Figure 4). Two-dimensional and 3D TEE should be used to monitor the guide sheath in real-time and ensure that the end of the guide is away from the inferior vena cava orifice and right atrial wall.

The Super Stiff guidewire can then be withdrawn and the adjustable delivery system (referred to as the delivery catheter) advanced to the end of the guide sheath. Once the delivery catheter reaches the end, the guide sheath is retrieved to the superior vena cava orifice.

**Figure 5:**
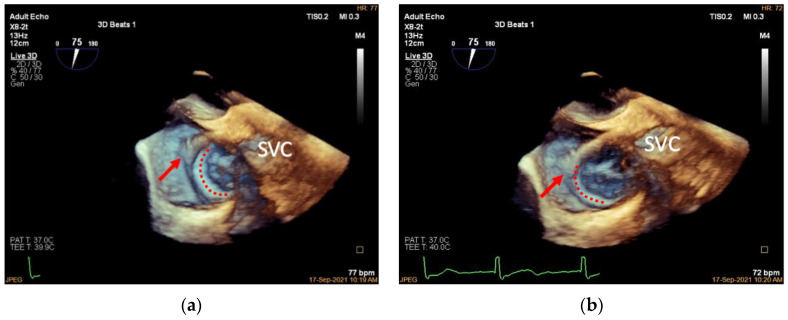
Adjusting the delivery catheter. (**a**): Three-dimensional adjusted mid-esophageal bicaval view (slight clockwise/counterclockwise rotation of the probe from the mid-esophageal bicaval view) demonstrating the head of the delivery catheter (red arrow) pointing into the right atrium above the anteroposterior TA junction (TA shown in red dotted line); (**b**): the same view showing the head of the delivery catheter pointing at the anteroposterior TA junction. SVC, superior vena cava.

The delivery catheter is adjusted under a 3D-adjusted mid-esophageal bicaval view. To obtain this view, the probe is rotated slightly clockwise/counterclockwise to obtain the appropriate depth required to view the TA using 3D imaging. The 3D volume is then manipulated to achieve better visualization of the posterior TA so that the catheter end is pointing at the target location (the anteroposterior commissure of TA, the middle of the posterior annulus, or the anterior 1/3 of the posterior annulus) (Figure 5).

### 3.3. Deployment of the Tapping Screw-Shaped Anchor

The tapping screw-shaped anchor is an essential part of the clip that is anchored into the target annular area first. It is designed to be pulled back during the subsequent clamping procedure and bring the adjacent annular tissue into the clip to be captured. This process achieves tissue plication and annular reduction.

Two TEE work planes are needed to deploy the anchor of the K-Clip™ system: the 3D TV en face view (referred to as the 3D work plane) and an X-Plane view (RV inflow–outflow view as the primary view, referred to as the X-Plane work plane). The X-Plane work plane imaging includes a ME RV inflow–outflow view that is usually around 60°, which is similar to that of an oblique four-chamber view. When the sample line is placed at the annular target location (the anteroposterior commissure of TA, the middle of the posterior annulus, or the anterior 1/3 of the posterior annulus), using the ME RV inflow–outflow view as the primary view will allow visualization of the posterior TA and the trajectory of the delivery catheter. More importantly, this work plane allows the assessment of annular tissue depth and monitoring of the anchor deployment. Note that although 3D TEE helps guide the delivery catheter to the target location, deployment of the tapping screw-shaped anchor should always be monitored under 2D TEE.

**Figure 6:**
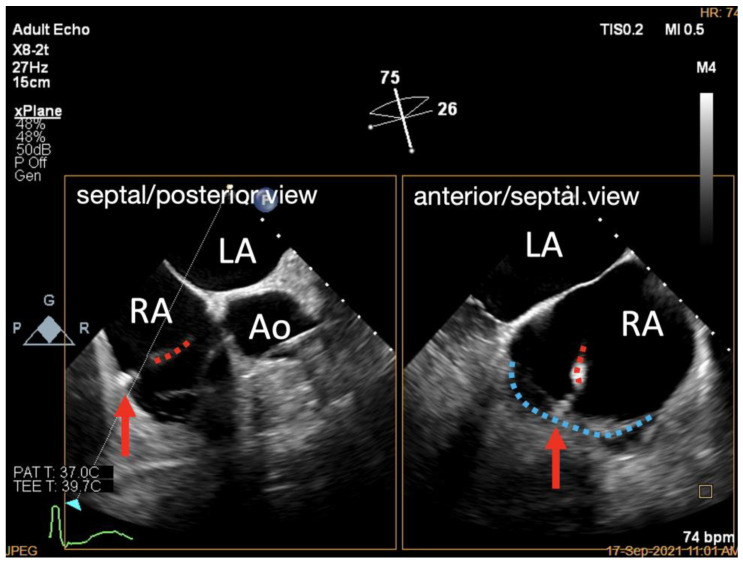
X-Plane work plane of K-Clip™ implantation. X-Plane work plane showing tapping screw (red arrow) anchored into the predetermined TA location (delivery catheter shown in red dotted line, posterior TA shown in blue dotted line). LA, left atrium; RA, right atrium; Ao, aorta.

**Figure 7:**
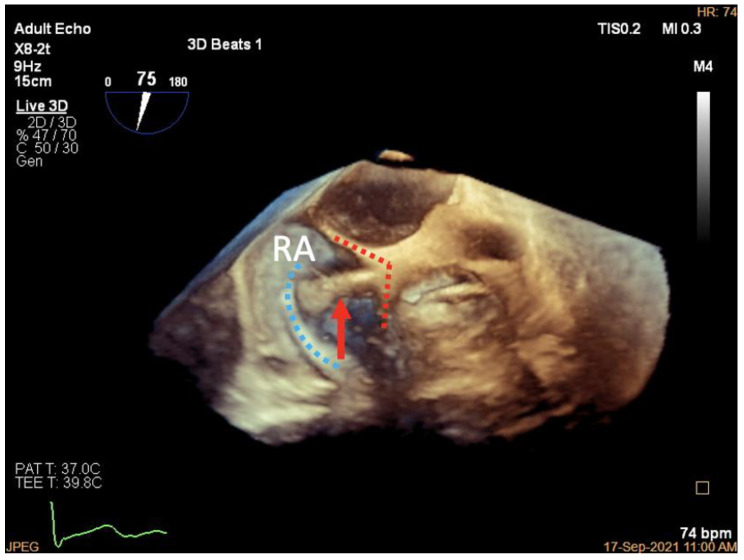
Three-dimensional work plane of K-Clip™ implantation. Three-dimensional work plane confirming tapping screw (red arrow) anchored at the target TA location (posterior TA shown in blue dotted line, clip arms shown in red dotted line). RA, right atrium.

Under the guidance of the X-Plane work plane, the delivery catheter is slowly advanced to bring the head of the device to the annular target and a tapping screw is anchored into the annulus (Figure 6). A pull-back test is then performed with 3D work plane monitoring to confirm that the tapping screw is deployed at the desired position (Figure 7).

### 3.4. Adjustment of the Clip Arm and Clamping of Annular Tissue

**Figure 8:**
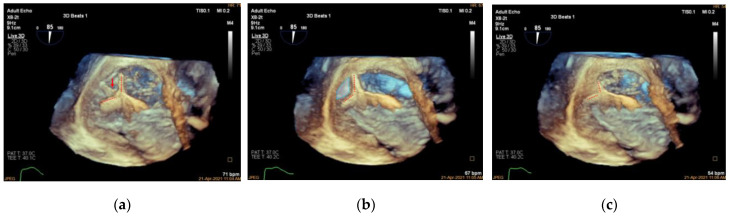
Clamping of annular tissue. (**a**): Three-dimensional work plane showing the clip arms aligned parallel to the tangent line with the tricuspid annulus (clip arms are shown as red dotted lines, anchor is indicated by a red arrow); (**b**): 3D work plane showing the target TA tissue is lifted and slowly clamped (blue area indicates lifted TA tissue); (**c**): 3D work plane showing the multilayer structure of the TA tissue and anchor clutched in the clip arms.

After the location of the tapping screw is verified as appropriate, the 3D work plane is used to guide the clip arms to open at the appropriate angle and rotate the delivery catheter so that the plane of the clip arms is at a cross-section with the target portion of the annulus. The tapping screw is then slowly withdrawn with the clip arms approaching the TA. As the surrounding annular tissue is brought into the clip arms, the arms can be closed to form a multilayer structure with the TA tissue and tapping screw being clutched within the clip arms (Figure 8).

### 3.5. Valve Function Assessment

**Figure 9:**
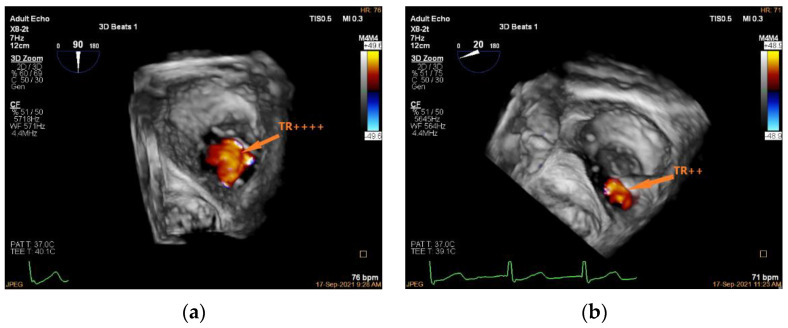
Three-dimensional CDI demonstrating TR jet before and after K-Clip™ implantation. (**a**): Three-dimensional CDI showing severe TR; (**b**): 3D CDI showing residual mild-moderate TR after transcatheter tricuspid annuloplasty. TR, tricuspid regurgitation.

**Figure 10:**
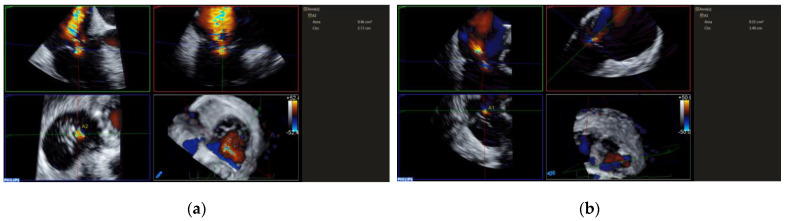
Measuring TR VCA before and after K-Clip™ implantation. (**a**): VCA showing severe TR; (**b**): VCA showing residual mild-moderate TR after transcatheter tricuspid annuloplasty.

Unlike edge-to-edge repair devices, an inflow gradient is seldom a concern after transcatheter annuloplasty, but residual TR should be carefully quantified using 2D and 3D CDI according to current guideline recommendations. Of note, 3D CDI could give qualitative implications regarding the degree of TR reduction and might be more reliable than 2D CDI as it does not rely on an imaging plane (Figure 9). However, qualitative evaluation is not recommended. The vena contracta area may provide reliable quantitative estimates of residual TR, but this approach is still under clinical investigation (Figure 10).

With several semi-quantitative and quantitative parameters proposed, all indices are still applicable for assessing transcatheter annuloplasty. As indicated in the current guideline, the wide range in severe TR prompts an extension of the severe TR category to include grades of “massive” and “torrential”, especially for patients undergoing transcatheter intervention. The SCOUT trial shows that TTVI achieves a significant reduction in mean vena contracta width, effective regurgitant orifice area by the proximal isovelocity surface area method, and quantitative Doppler, but the residual TR remains severe regarding these quantitative parameters. The change in TR grade after implantation of the K-Clip™ system is presented in Figure S3. In our initial experience, 36.9% of patients achieved a two-grade reduction in TR severity, and 31.6% of patients achieved a one-grade reduction in TR severity. However, a significant minority (36.9%) still had severe TR.

If there is no satisfactory reduction in TR, then the clip arms can be opened, and the TA can be re-targeted and re-clamped. However, a second clip implantation has not yet been attempted as it involves the residual size of the TA and the site of the second clip.

**Figure 11:**
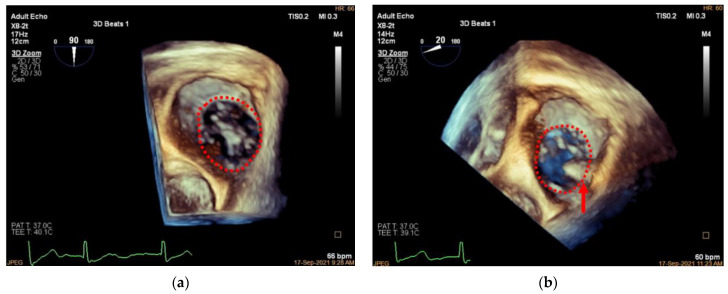
Three-dimensional comparison of TA morphology before and after K-Clip™ implantation. (**a**): Three-dimensional TEE work plane showing the TA morphology before annuloplasty; (**b**): the same view showing the TA (red dotted line) and clip position (red arrow) after intervention.

**Figure 12:**
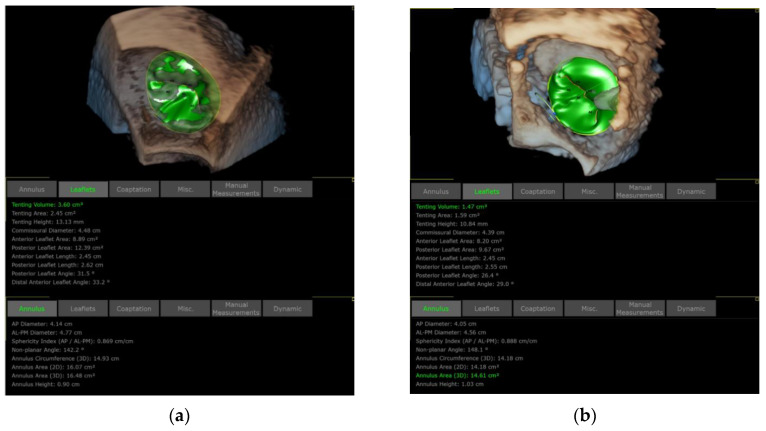
Comparison of TA and TV dimensions before and after K-Clip™ implantation. (**a**): TA and TV measurements before annuloplasty; (**b**): TA and TV measurements after intervention.

Three-dimensional TEE is also applied to assess the TA and TV morphology including: (1) anteroposterior and left–right diameters of TA and (2) TA circumference and area (Figure 11 and Figure 12). In our initial experience, the TA perimeter and area reduced from 14.13 ± 1.35 mm and 14.93 ± 2.60 mm^2^ to 12.83 ± 1.47 mm and 12.40 ± 2.61 mm^2^, respectively, immediately after the procedure (both *p* < 0.001).

### 3.6. Assessment of Clutch Status

The clip position and stability should be verified using both 2D and 3D TEE. After this verification, the clip is locked, and the guide and delivery sheath can be withdrawn. The clip stability is determined both visually and indirectly by calculating the annular reduction length. The clip should only slightly move perpendicularly to the annular plane under visual assessment, and the annular reduction length (the difference between the TA circumferences before and after clamping) should not be significantly shorter than the designed value of the device (25, 28, 32, and 36 mm).

Though not part of an evaluation for the procedural success, a follow-up on the right chambers of the heart would provide critical information. Neither regression of RV diameter nor improvement in its function (either fractional area changes or tricuspid annular plane systolic excursion) was observed at a one-month follow-up post-procedure (all *p* > 0.05, paired *t*-test), but RA area was significantly reduced (*p* = 0.008, paired *t*-test) from 33.46 ± 6.77 mm^2^ to 27.86 ± 3.89 mm^2^. Long-term follow-up on these parameters is needed.

The proposed protocol, summarized in Table 1, is designed for teams that are beginning and/or learning how to perform the K-Clip™ implantation to reduce the time needed for the proficiency of the echocardiographers and the interventionists. The simplified TEE monitoring regimen is not a substitute for the comprehensive knowledge and imaging protocols covered in the guidelines.

## 4. Pitfalls and Potential Main Errors

The K-Clip™ tapping screw-shaped anchor should ideally be inserted towards the leaflet hinge point. If inserted too close to the atrial side, the anchor might perforate the atrial wall, and when pulled back, it may bring atrial tissue rather than annular tissue into the clip, which may not result in an effective annular reduction. On the other hand, if inserted too close to the leaflet side, the anchor may penetrate the leaflet and cause a leaflet tear when pulled back.

A common pitfall of K-Clip™ implantation is inserting the anchor parallel to the annular plane without any angulation. Such practice will likely cause the anchor to slip towards the atrial side upon the trigger and deviate from the pre-determined target location. Moreover, slight tilting of the anchor towards the ventricle side would help bring more annular tissue rather than atrial tissue into the clamp when it is pulled back. The angulation of the tapping screw can be best observed with the proposed 2D X-plane work plane. This 2D TEE view could also be used intermittently during the clamping to verify that the annular tissue, rather than atrial or valve tissue, will be clamped.

A potential main error of K-Clip™ implantation is the imperfect alignment of the clip arms with the annulus, which would lead to clamping of valve tissue and less or no annular reduction. The proposed 3D en face view should be used to ensure that the plane of the clip arms is at a cross-section with the target portion of the annulus. If an imperfect alignment does happen, then the clip arms can be opened, and the TA can be re-clamped.

Another potential main error of K-Clip™ implantation is its interference with the right coronary artery. Therefore, a guide wire should be placed into this branch before implantation in case any further intervention is needed. A coronary angiogram should be performed immediately after the implantation.

## 5. Conclusions

This review describes a systematic 2D and 3D TEE guidance protocol for transcatheter tricuspid annuloplasty with the novel K-Clip™ system. It is the first overview and analysis of an imaging guidance protocol for the K-Clip™ annular clamping device, which is tailored to the unique mechanism of this repair system. This protocol, which entails only several standard 2D and 3D views as recommended by the current guidelines, should provide a steeper learning curve for echocardiographers and interventionalists while simplifying the steps for implantation.

## Figures and Tables

**Table 1:** Summary of the echocardiographic protocols for the step-by-step guidance of K-Clip implantation.

Step	TEE Views	Angle Ranges	Notes
Step 1. Introducing the delivery system	2D and 3D mid-esophageal bicaval view	70–110°	
	3D adjusted mid-esophageal bicaval view	70–110°	Slight clockwise/counter-clockwise rotation of the probe from the mid-esophageal bicaval view for appropriate depth to visualize the TA
Step 2. Deployment of the tapping screw-shaped anchor	X-plane view with ME RV inflow–outflow view as the primary view	about 60°	Deployment of the tapping screw-shaped anchor should always be monitored under 2D TEE
	3D TV en face view	about 60°	Further confirmation of the target annular location
Step 3. Adjustment of the clip arm and clamping of annular tissue	3D TV en face view	about 60°	
Step 4. Valve function assessment	2D and 3D (preferable) color Doppler imaging	Multiple views required	3D color Doppler imaging is preferred as tricuspid intervention results in highly complex valve geometry
Step 5. Assessment of clutch status	X-plane view with ME RV inflow–outflow view as the primary view	about 60°	Clip stability is preferably verified under 2D imaging due to its high resolution
	3D TV en face view	Any angle permitting acquisition of the whole TA volume would be acceptable	

## Data Availability

Details of the first-in-human (FIM) study of the K-Clip device are included in the Supplementary Material.

## Supplementary Materials

The following supporting information can be downloaded at: https://www.mdpi.com/article/10.3390/jcdd9120415/s1, Figure S1: Patient screening protocol for the first-in-human study of the K-ClipTM system; Figure S2: NYHA functional status was improved after implantation of the K-ClipTM system; Figure S3: TR was reduced immediately and one-month post-procedure; Table S1: Baseline demographics of patients who received K-Clip implantation; Table S2: Baseline characteristics of tricuspid regurgitation by echocardiography; Table S3: Summary of the procedural safety of K-Clip implantation.
