# Supplementary material for: Real-Time Monitoring and Step-by-Step Guidance for Transcatheter Tricuspid Annuloplasty Using Transesophageal Echocardiography
Source: J Cardiovasc Dev Dis. 2022 Nov 25;9(12):415. doi: 10.3390/jcdd9120415 (PMC9781931; doi:10.3390/jcdd9120415)
Supplement: Supplementary file 1 [file jcdd-09-00415-s001.zip › jcdd-2022088-supplementary.pdf]

Journal of
Cardiovascular Development and Disease

MDPI

Supplementary Materials

Real-Time Monitoring and Step-by-Step Guidance for Transcatheter Tricuspid Annuloplasty Using Transesophageal Echocardiography

Yu Liu, Wei Li, Daxin Zhou, Xiaochun Zhang, Dehong Kong, Zhenyi Ge, Haiyan Chen, Xianhong Shu, Cuizhen Pan and Junbo Ge

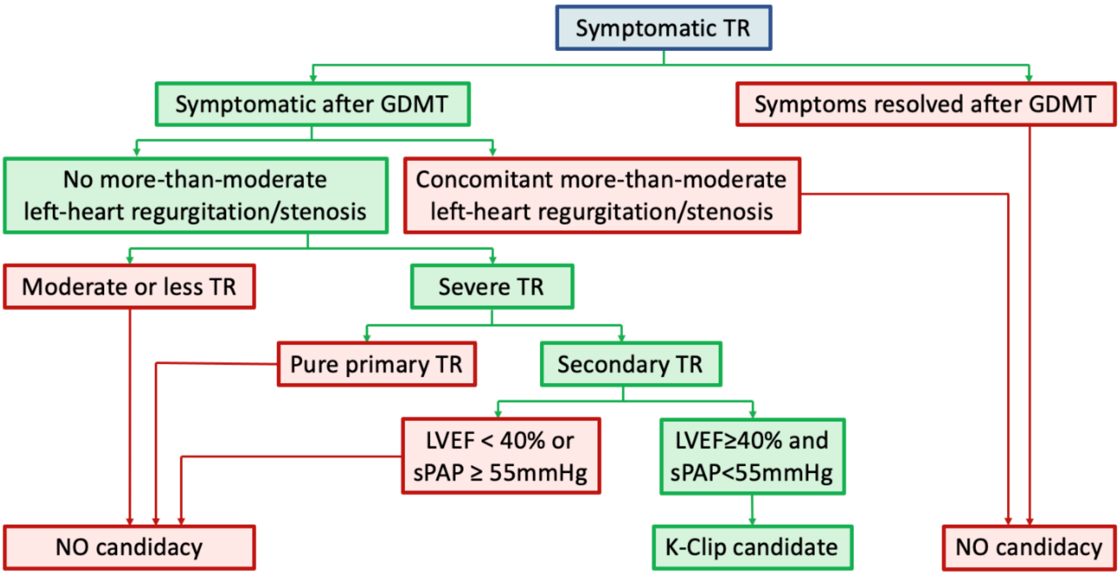

Figure S1. Patient screening protocol for the First-in-human study of K-Clip™ system. TR, tricuspid regurgitation; GDMT, guideline-directed medical therapy; LVEF, left ventricular ejection fraction; sPAP, systolic pulmonary arterial pressure.

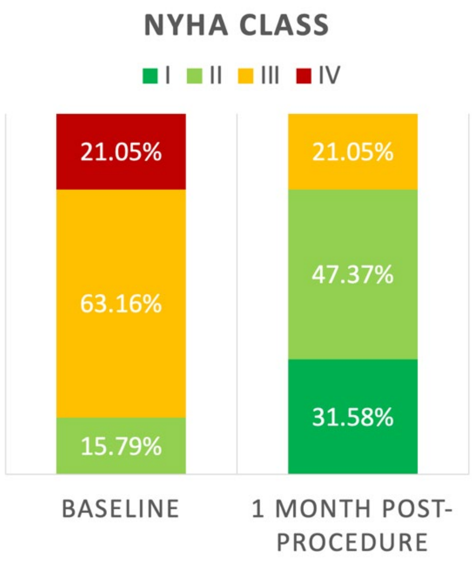

Figure S2. NYHA functional status was improved after implantation of the K-Clip™ system. NYHA, New York Heart Association.

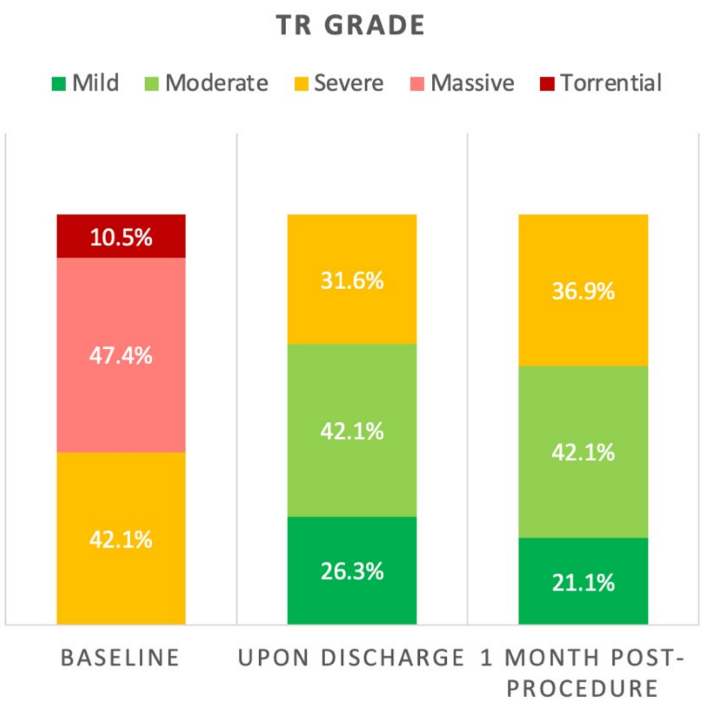

Figure S3. TR was reduced immediately and 1-month post-procedure. TR, tricuspid regurgitation.

Table S1. Baseline demographics of patients received K-Clip implantation.

**Variable**	***N*=19**
Age	73.4±8.7
Gender (Male)	63.2%
NYHA Class	
I	0
II	3(15.8%)
III	12(63.2%)
IV	4(21.1%)
Hypertension	9(47.3%)
Diabetes	4(21.1%)
Atrial fibrillation (AF)	15(78.9%)
Previous mitral valve surgery	4(21.1%)
I-II degree atrioventricular block	0
Myocardial infarction	0
Stroke	0
Percutaneous cardiac intervention	1(5.2%)
ICD	2(10.5%)
Renal failure	0
Previous annulus intervention	0
STS score, %	7.9±3.1

NYHA, New York Heart Association; ICD, intra-cardiac defibrillator; STS, society of thoracic surgeons.

Table S2. Baseline characteristics of tricuspid regurgitation by echocardiography.

**Variable**	***N*=19**
TR grade	
Severe	8(42.1%)
Massive	9(47.3%)
Torrential	2(10.5%)
TR etiology	
Functional	15(78.9%)
Secondary to AF	11(57.8%)
Secondary to left heart disease	4(21.1%)
ICD-lead related	0
Degenerative	0
Mixed etiology	4(21.1%)
Right atrium (major*minor axis), mm	(72±10.8)*(43.8±7.3)
Right ventricle (basal diameter), mm	48±9
Tricuspid annulus diameter (major*minor axis), mm	(47.2±5.0)*(43.8±7.3)
Vena contracta width (major*minor axis), mm	(15.3+5.9)*(14.8±5.4)

TR, tricuspid regurgitation; AF, atrial fibrillation; ICD, intra-cardiac defibrillator.*, means "times".

Table S3. Summary of the procedural safety of K-Clip implantation.

Adverse Events	*N*=19	
	**Event counts**	**Subject counts (%)**
All adverse events (AE)	3	2 (10.53)
AE during screening	0	0
AE during treatment	3	2 (10.53)
Relation to test device		
Related	1	1(5.26)
Not related	2	1(5.26)
Severity of AE		
Mild	3	2 (10.53)
Moderate	0	0
Severe	0	0
All severe adverse events (SAE)		
SAE during screening	0	0
SAE during treatment	1	1(5.26)
Relation to test device		
Related	0	0
Not related	1	1(5.26)
SAE leading to death	0	0
